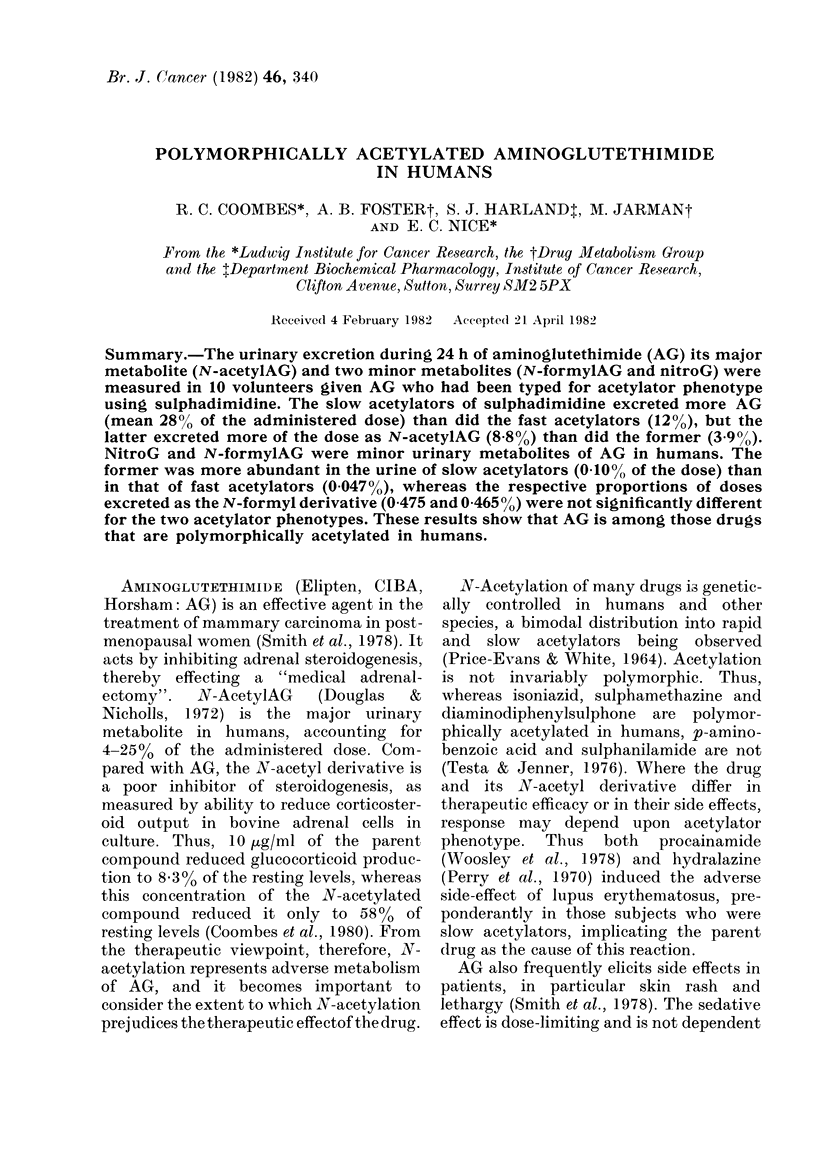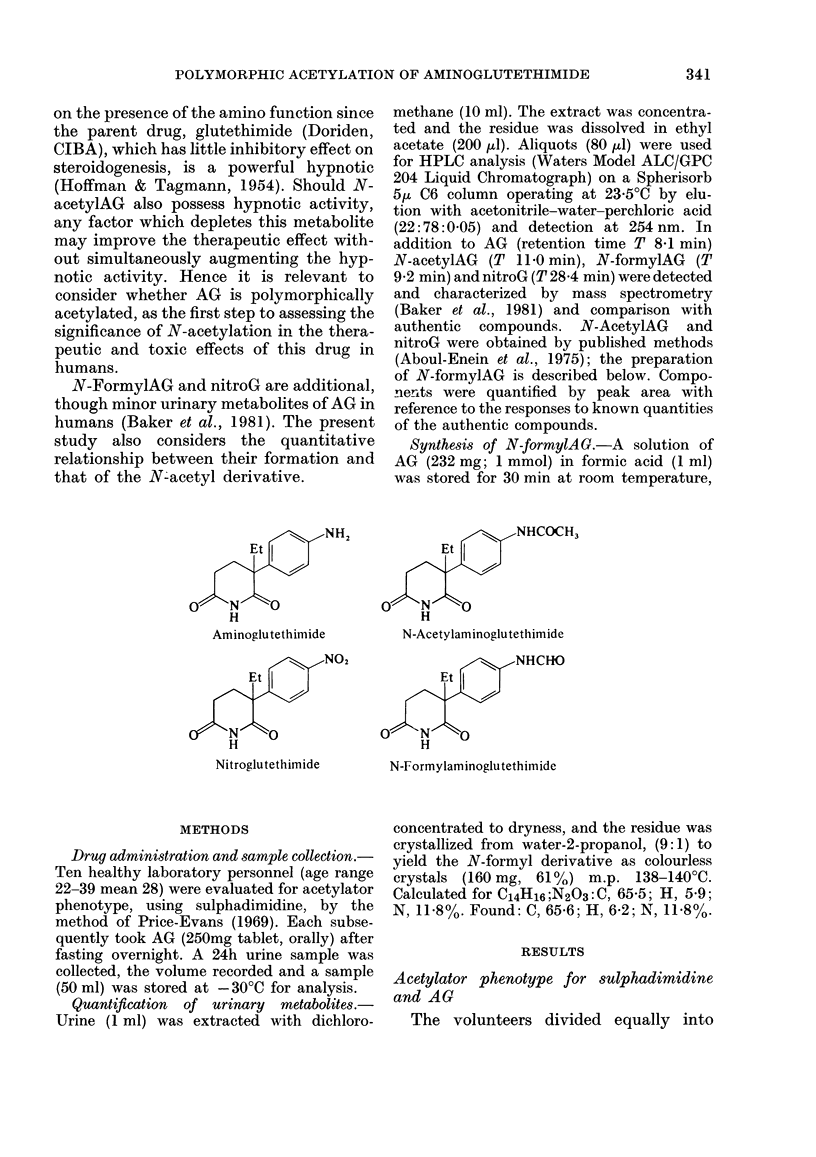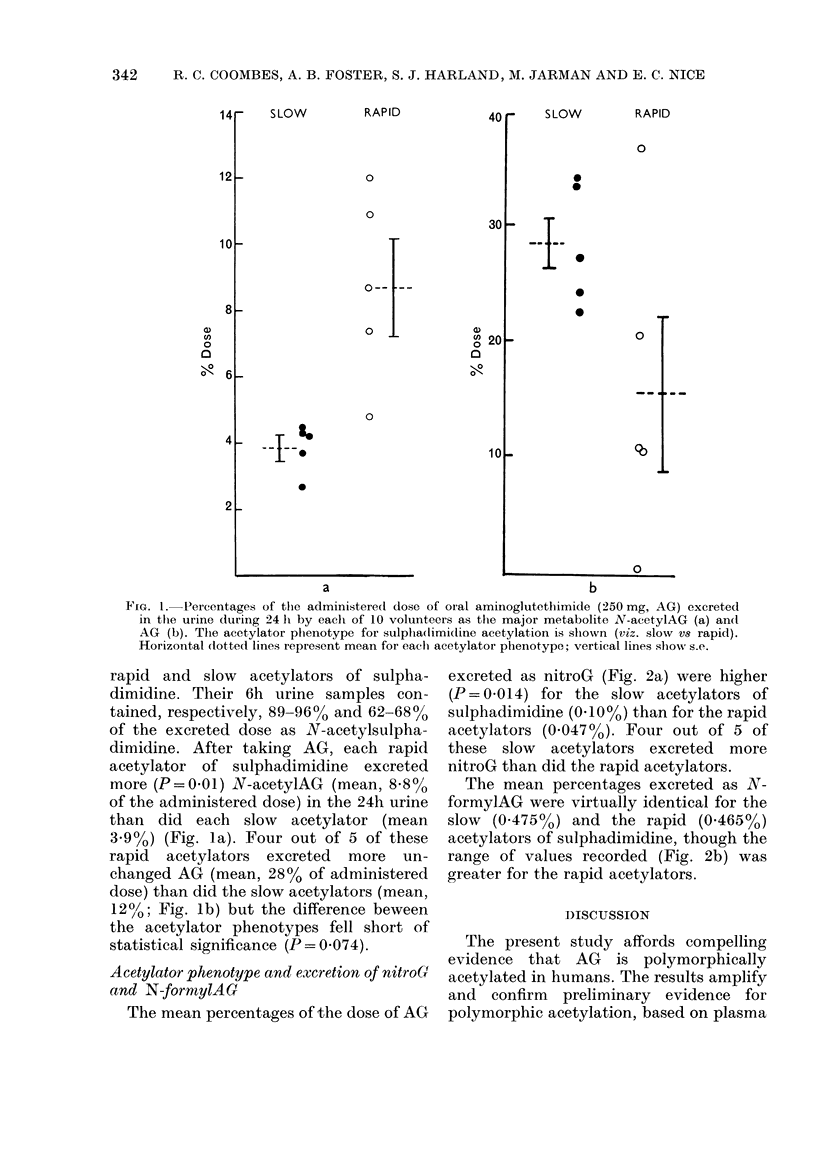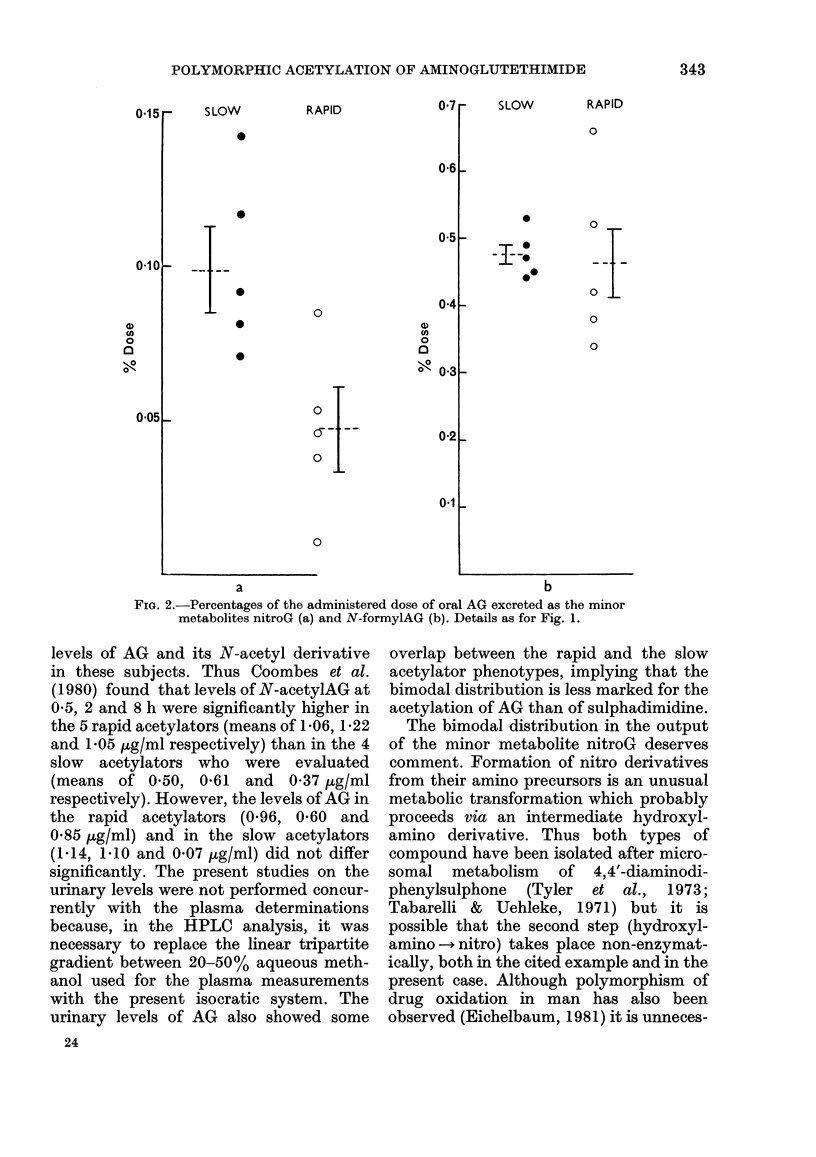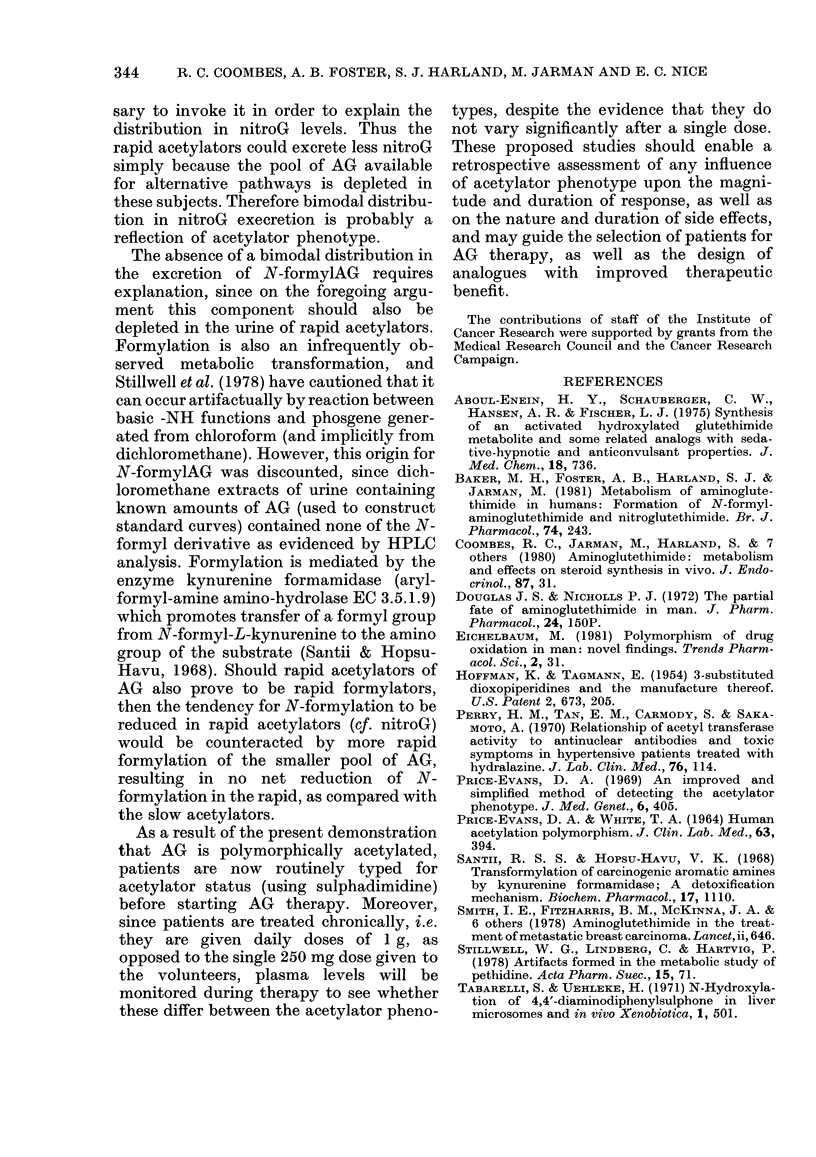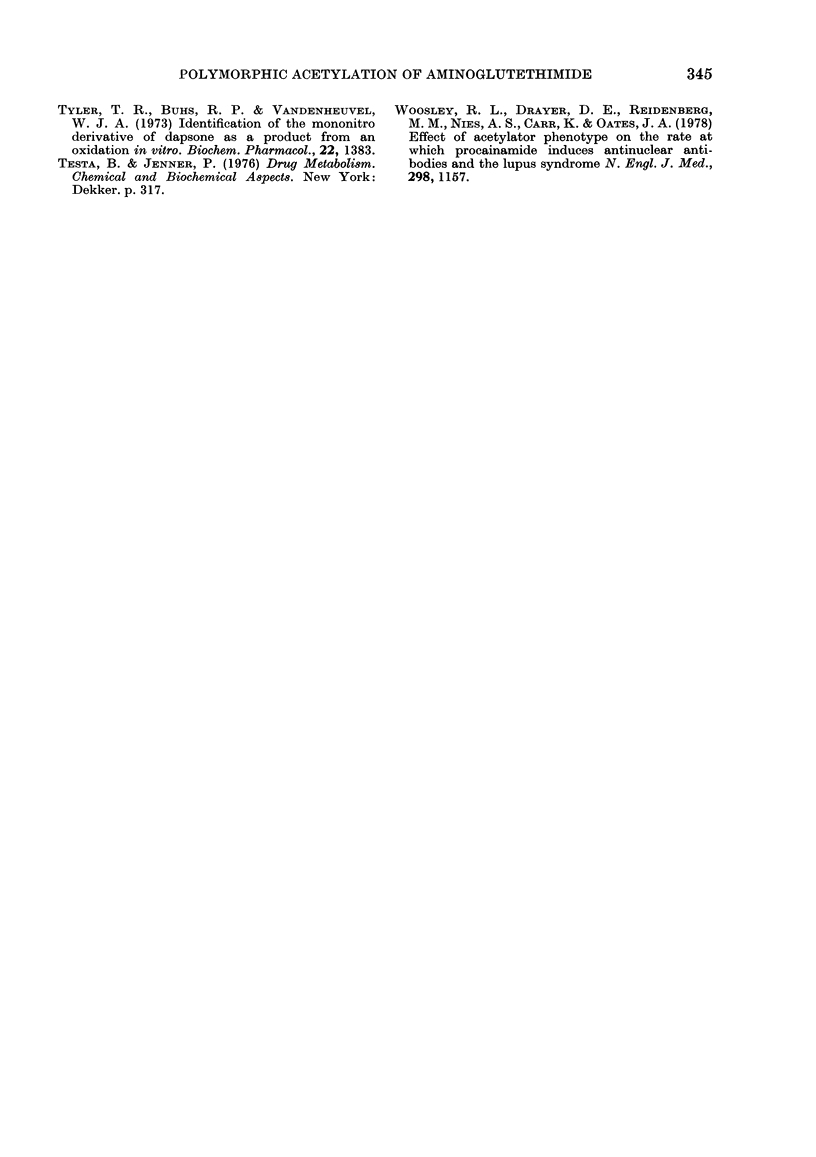# Polymorphically acetylated aminoglutethimide in humans.

**DOI:** 10.1038/bjc.1982.209

**Published:** 1982-09

**Authors:** R. C. Coombes, A. B. Foster, S. J. Harland, M. Jarman, E. C. Nice

## Abstract

The urinary excretion during 24 h of aminoglutethimide (AG) its major metabolite (N-acetylAG) and two minor metabolites (N-formylAG and nitroG) were measured in 10 volunteers given AG who had been typed for acetylator phenotype using sulphadimidine. The slow acetylators of sulphadimidine excreted more AG (mean 28% of the administered dose) than did the fast acetylators (12%), but the latter excreted more of the dose as N-acetylAG (8.8%) than did the former (3.9%). NitroG and N-formylAG were minor urinary metabolites of AG in humans. The former was more abundant in the urine of slow acetylators (0.10% of the dose) than in that of fast acetylators (0.047%), whereas the respective proportions of doses excreted as the N-formyl derivative (0.475 and 0.465%) were not significantly different for the two acetylator phenotypes. These results show that AG is among those drugs that are polymorphically acetylated in humans.


*Br. J. Cancer* (1982) **46**, 340

### POLYMORPHICALLY ACETYLATED AMINOGLUTETHIMIDE IN HUMANS

R. C. COOMBES*, A. B. FOSTER†, S. J. HARLAND‡, M. JARMAN†
AND E. C. NICE*

*From the *Ludwig Institute for Cancer Research, the †Drug Metabolism Group and the ‡Department Biochemical Pharmacology, Institute of Cancer Research, Clifton Avenue, Sutton, Surrey SM2 5PX*

Received 4 February 1982 Accepted 21 April 1982

**Summary.—The urinary excretion during 24 h of aminoglutethimide (AG) its major metabolite (*N*-acetylAG) and two minor metabolites (*N*-formylAG and nitroG) were measured in 10 volunteers given AG who had been typed for acetylator phenotype using sulphadimidine. The slow acetylators of sulphadimidine excreted more AG (mean 28% of the administered dose) than did the fast acetylators (12%), but the latter excreted more of the dose as *N*-acetylAG (8·8%) than did the former (3·9%). NitroG and *N*-formylAG were minor urinary metabolites of AG in humans. The former was more abundant in the urine of slow acetylators (0·10% of the dose) than in that of fast acetylators (0·047%), whereas the respective proportions of doses excreted as the *N*-formyl derivative (0·475 and 0·465%) were not significantly different for the two acetylator phenotypes. These results show that AG is among those drugs that are polymorphically acetylated in humans.**

AMINOGLUTETHIMIDE (Elipten, CIBA, Horsham: AG) is an effective agent in the treatment of mammary carcinoma in post-menopausal women (Smith *et al.*, 1978). It acts by inhibiting adrenal steroidogenesis, thereby effecting a "medical adrenalectomy". *N*-AcetylAG (Douglas & Nicholls, 1972) is the major urinary metabolite in humans, accounting for 4–25% of the administered dose. Compared with AG, the *N*-acetyl derivative is a poor inhibitor of steroidogenesis, as measured by ability to reduce corticosteroid output in bovine adrenal cells in culture. Thus, 10 μg/ml of the parent compound reduced glucocorticoid production to 8·3% of the resting levels, whereas this concentration of the *N*-acetylated compound reduced it only to 58% of resting levels (Coombes *et al.*, 1980). From the therapeutic viewpoint, therefore, *N*-acetylation represents adverse metabolism of AG, and it becomes important to consider the extent to which *N*-acetylation prejudices the therapeutic effect of the drug.

*N*-Acetylation of many drugs is genetically controlled in humans and other species, a bimodal distribution into rapid and slow acetylators being observed (Price-Evans & White, 1964). Acetylation is not invariably polymorphic. Thus, whereas isoniazid, sulphamethazine and diaminodiphenylsulphone are polymorphically acetylated in humans, *p*-aminobenzoic acid and sulphanilamide are not (Testa & Jenner, 1976). Where the drug and its *N*-acetyl derivative differ in therapeutic efficacy or in their side effects, response may depend upon acetylator phenotype. Thus both procainamide (Woosley *et al.*, 1978) and hydralazine (Perry *et al.*, 1970) induced the adverse side-effect of lupus erythematosus, preponderantly in those subjects who were slow acetylators, implicating the parent drug as the cause of this reaction.

AG also frequently elicits side effects in patients, in particular skin rash and lethargy (Smith *et al.*, 1978). The sedative effect is dose-limiting and is not dependent on the presence of the amino function since the parent drug, glutethimide (Doriden, CIBA), which has little inhibitory effect on steroidogenesis, is a powerful hypnotic (Hoffman & Tagmann, 1954). Should *N*-acetylAG also possess hypnotic activity, any factor which depletes this metabolite may improve the therapeutic effect without simultaneously augmenting the hypnotic activity. Hence it is relevant to consider whether AG is polymorphically acetylated, as the first step to assessing the significance of *N*-acetylation in the therapeutic and toxic effects of this drug in humans.

*N*-FormylAG and nitroG are additional, though minor urinary metabolites of AG in humans (Baker *et al.*, 1981). The present study also considers the quantitative relationship between their formation and that of the *N*-acetyl derivative.

Aminoglutethimide

N-Acetylaminoglutethimide

Nitroglutethimide

N-Formylaminoglutethimide

### METHODS

*Drug administration and sample collection.*—Ten healthy laboratory personnel (age range 22–39 mean 28) were evaluated for acetylator phenotype, using sulphadimidine, by the method of Price-Evans (1969). Each subsequently took AG (250mg tablet, orally) after fasting overnight. A 24h urine sample was collected, the volume recorded and a sample (50 ml) was stored at −30°C for analysis.

*Quantification of urinary metabolites.*—Urine (1 ml) was extracted with dichloromethane (10 ml). The extract was concentrated and the residue was dissolved in ethyl acetate (200 μl). Aliquots (80 μl) were used for HPLC analysis (Waters Model ALC/GPC 204 Liquid Chromatograph) on a Spherisorb 5μ C6 column operating at 23·5°C by elution with acetonitrile–water–perchloric acid (22:78:0·05) and detection at 254 nm. In addition to AG (retention time *T* 8·1 min) *N*-acetylAG (*T* 11·0 min), *N*-formylAG (*T* 9·2 min) and nitroG (*T* 28·4 min) were detected and characterized by mass spectrometry (Baker *et al.*, 1981) and comparison with authentic compounds. *N*-AcetylAG and nitroG were obtained by published methods (Aboul-Enein *et al.*, 1975); the preparation of *N*-formylAG is described below. Components were quantified by peak area with reference to the responses to known quantities of the authentic compounds.

*Synthesis of N-formylAG.*—A solution of AG (232 mg; 1 mmol) in formic acid (1 ml) was stored for 30 min at room temperature, concentrated to dryness, and the residue was crystallized from water-2-propanol, (9:1) to yield the *N*-formyl derivative as colourless crystals (160 mg, 61%) m.p. 138–140°C. Calculated for $C_{14}H_{16}$;$N_2O_3$:C, 65·5; H, 5·9; N, 11·8%. Found: C, 65·6; H, 6·2; N, 11·8%.

### RESULTS

### *Acetylator phenotype for sulphadimidine and AG*

The volunteers divided equally into

FIG. 1.—Percentages of the administered dose of oral aminoglutethimide (250 mg, AG) excreted in the urine during 24 h by each of 10 volunteers as the major metabolite *N*-acetylAG (a) and AG (b). The acetylator phenotype for sulphadimidine acetylation is shown (*viz.* slow *vs* rapid). Horizontal dotted lines represent mean for each acetylator phenotype; vertical lines show s.e.

rapid and slow acetylators of sulphadimidine. Their 6h urine samples contained, respectively, 89–96% and 62–68% of the excreted dose as *N*-acetylsulphadimidine. After taking AG, each rapid acetylator of sulphadimidine excreted more ($P = 0{\cdot}01$) *N*-acetylAG (mean, 8·8% of the administered dose) in the 24h urine than did each slow acetylator (mean 3·9%) (Fig. 1a). Four out of 5 of these rapid acetylators excreted more unchanged AG (mean, 28% of administered dose) than did the slow acetylators (mean, 12%; Fig. 1b) but the difference beween the acetylator phenotypes fell short of statistical significance ($P = 0{\cdot}074$).

*Acetylator phenotype and excretion of nitroG and* N-*formylAG*

The mean percentages of the dose of AG excreted as nitroG (Fig. 2a) were higher ($P = 0{\cdot}014$) for the slow acetylators of sulphadimidine (0·10%) than for the rapid acetylators (0·047%). Four out of 5 of these slow acetylators excreted more nitroG than did the rapid acetylators.

The mean percentages excreted as *N*-formylAG were virtually identical for the slow (0·475%) and the rapid (0·465%) acetylators of sulphadimidine, though the range of values recorded (Fig. 2b) was greater for the rapid acetylators.

### DISCUSSION

The present study affords compelling evidence that AG is polymorphically acetylated in humans. The results amplify and confirm preliminary evidence for polymorphic acetylation, based on plasma

FIG. 2.—Percentages of the administered dose of oral AG excreted as the minor metabolites nitroG (a) and *N*-formylAG (b). Details as for Fig. 1.

levels of AG and its *N*-acetyl derivative in these subjects. Thus Coombes *et al.* (1980) found that levels of *N*-acetylAG at 0·5, 2 and 8 h were significantly higher in the 5 rapid acetylators (means of 1·06, 1·22 and 1·05 μg/ml respectively) than in the 4 slow acetylators who were evaluated (means of 0·50, 0·61 and 0·37 μg/ml respectively). However, the levels of AG in the rapid acetylators (0·96, 0·60 and 0·85 μg/ml) and in the slow acetylators (1·14, 1·10 and 0·07 μg/ml) did not differ significantly. The present studies on the urinary levels were not performed concurrently with the plasma determinations because, in the HPLC analysis, it was necessary to replace the linear tripartite gradient between 20–50% aqueous methanol used for the plasma measurements with the present isocratic system. The urinary levels of AG also showed some overlap between the rapid and the slow acetylator phenotypes, implying that the bimodal distribution is less marked for the acetylation of AG than of sulphadimidine.

The bimodal distribution in the output of the minor metabolite nitroG deserves comment. Formation of nitro derivatives from their amino precursors is an unusual metabolic transformation which probably proceeds *via* an intermediate hydroxylamino derivative. Thus both types of compound have been isolated after microsomal metabolism of 4,4′-diaminodiphenylsulphone (Tyler *et al.*, 1973; Tabarelli & Uehleke, 1971) but it is possible that the second step (hydroxylamino → nitro) takes place non-enzymatically, both in the cited example and in the present case. Although polymorphism of drug oxidation in man has also been observed (Eichelbaum, 1981) it is unnecessary to invoke it in order to explain the distribution in nitroG levels. Thus the rapid acetylators could excrete less nitroG simply because the pool of AG available for alternative pathways is depleted in these subjects. Therefore bimodal distribution in nitroG execretion is probably a reflection of acetylator phenotype.

The absence of a bimodal distribution in the excretion of *N*-formylAG requires explanation, since on the foregoing argument this component should also be depleted in the urine of rapid acetylators. Formylation is also an infrequently observed metabolic transformation, and Stillwell *et al.* (1978) have cautioned that it can occur artifactually by reaction between basic -NH functions and phosgene generated from chloroform (and implicitly from dichloromethane). However, this origin for *N*-formylAG was discounted, since dichloromethane extracts of urine containing known amounts of AG (used to construct standard curves) contained none of the *N*-formyl derivative as evidenced by HPLC analysis. Formylation is mediated by the enzyme kynurenine formamidase (arylformyl-amine amino-hydrolase EC 3.5.1.9) which promotes transfer of a formyl group from *N*-formyl-*L*-kynurenine to the amino group of the substrate (Santii & Hopsu-Havu, 1968). Should rapid acetylators of AG also prove to be rapid formylators, then the tendency for *N*-formylation to be reduced in rapid acetylators (*cf.* nitroG) would be counteracted by more rapid formylation of the smaller pool of AG, resulting in no net reduction of *N*-formylation in the rapid, as compared with the slow acetylators.

As a result of the present demonstration that AG is polymorphically acetylated, patients are now routinely typed for acetylator status (using sulphadimidine) before starting AG therapy. Moreover, since patients are treated chronically, *i.e.* they are given daily doses of 1 g, as opposed to the single 250 mg dose given to the volunteers, plasma levels will be monitored during therapy to see whether these differ between the acetylator phenotypes, despite the evidence that they do not vary significantly after a single dose. These proposed studies should enable a retrospective assessment of any influence of acetylator phenotype upon the magnitude and duration of response, as well as on the nature and duration of side effects, and may guide the selection of patients for AG therapy, as well as the design of analogues with improved therapeutic benefit.

The contributions of staff of the Institute of Cancer Research were supported by grants from the Medical Research Council and the Cancer Research Campaign.

### REFERENCES

ABOUL-ENEIN, H. Y., SCHAUBERGER, C. W., HANSEN, A. R. & FISCHER, L. J. (1975) Synthesis of an activated hydroxylated glutethimide metabolite and some related analogs with sedative-hypnotic and anticonvulsant properties. *J. Med. Chem.*, **18**, 736.

BAKER, M. H., FOSTER, A. B., HARLAND, S. J. & JARMAN, M. (1981) Metabolism of aminoglutethimide in humans: Formation of *N*-formylaminoglutethimide and nitroglutethimide. *Br. J. Pharmacol.*, **74**, 243.

COOMBES, R. C., JARMAN, M., HARLAND, S. & 7 others (1980) Aminoglutethimide: metabolism and effects on steroid synthesis in vivo. *J. Endocrinol.*, **87**, 31.

DOUGLAS J. S. & NICHOLLS P. J. (1972) The partial fate of aminoglutethimide in man. *J. Pharm. Pharmacol.*, **24**, 150P.

EICHELBAUM, M. (1981) Polymorphism of drug oxidation in man: novel findings. *Trends Pharmacol. Sci.*, **2**, 31.

HOFFMAN, K. & TAGMANN, E. (1954) 3-substituted dioxopiperidines and the manufacture thereof. *U.S. Patent* 2, 673, 205.

PERRY, H. M., TAN, E. M., CARMODY, S. & SAKAMOTO, A. (1970) Relationship of acetyl transferase activity to antinuclear antibodies and toxic symptoms in hypertensive patients treated with hydralazine. *J. Lab. Clin. Med.*, **76**, 114.

PRICE-EVANS, D. A. (1969) An improved and simplified method of detecting the acetylator phenotype. *J. Med. Genet.*, **6**, 405.

PRICE-EVANS, D. A. & WHITE, T. A. (1964) Human acetylation polymorphism. *J. Clin. Lab. Med.*, **63**, 394.

SANTII, R. S. S. & HOPSU-HAVU, V. K. (1968) Transformylation of carcinogenic aromatic amines by kynurenine formamidase; A detoxification mechanism. *Biochem. Pharmacol.*, **17**, 1110.

SMITH, I. E., FITZHARRIS, B. M., MCKINNA, J. A. & 6 others (1978) Aminoglutethimide in the treatment of metastatic breast carcinoma. *Lancet*, ii, 646.

STILLWELL, W. G., LINDBERG, C. & HARTVIG, P. (1978) Artifacts formed in the metabolic study of pethidine. *Acta Pharm. Suec.*, **15**, 71.

TABARELLI, S. & UEHLEKE, H. (1971) N-Hydroxylation of 4,4′-diaminodiphenylsulphone in liver microsomes and *in vivo Xenobiotica*, **1**, 501.

TYLER, T. R., BUHS, R. P. & VANDENHEUVEL, W. J. A. (1973) Identification of the mononitro derivative of dapsone as a product from an oxidation *in vitro*. *Biochem. Pharmacol.*, **22**, 1383.

TESTA, B. & JENNER, P. (1976) *Drug Metabolism. Chemical and Biochemical Aspects*. New York: Dekker. p. 317.

WOOSLEY, R. L., DRAYER, D. E., REIDENBERG, M. M., NIES, A. S., CARR, K. & OATES, J. A. (1978) Effect of acetylator phenotype on the rate at which procainamide induces antinuclear antibodies and the lupus syndrome *N. Engl. J. Med.*, **298**, 1157.